# Regulation mechanism of cooking duration on flavor compounds in squid tentacles: dynamic contributions of free amino acids, fatty acids, and minerals

**DOI:** 10.3389/fnut.2025.1695733

**Published:** 2026-01-29

**Authors:** Xu Dan, Zhu Jian, Zeng Junjie, Fang Yi, Deng Shanggui, Yu Haixia, Zhang Xiaojun

**Affiliations:** 1 Zhejiang Marine Fisheries Research Institute, Zhoushan, Zhejiang, China; 2 College of Food and Medicine, Zhejiang Ocean University, Zhoushan, Zhejiang, China; 3 Ocean Research Center of Zhoushan, Zhejiang University, Zhoushan, Zhejiang, China

**Keywords:** cooking duration, dynamic contributions, fatty acids, flavor compounds, free amino acids, minerals, sensory evaluation, squid tentacles

## Abstract

**Background:**

The unique flavors of squid tentacles are developed during traditional stir-frying, where various flavor compounds interact dynamically. This study investigates the impact of cooking duration on flavor compound formation, focusing on the roles of free amino acids (FAAs), fatty acids (FFAs), and minerals.

**Methods:**

Argentine squid tentacles (*Illex argentinus*) were stir-fried at 120°C for 0, 2, 5, 10, and 15 min. Mineral content (Na, Mg, K, Fe, Zn, Ca) was quantified according to national standards. FAAs were extracted using trichloroacetic acid and analyzed. FFAs were determined by gas chromatography. Taste attributes were assessed using an electronic tongue and sensory evaluation by a trained panel. Statistical analyses included principal component analysis (PCA) and correlation analysis, with significance tested using the least significant difference (LSD) method (*P* < 0.05).

**Results:**

Stir-frying duration significantly affected the accumulation of FAAs, particularly glutamic acid and aspartic acid, which are key contributors to umami taste. Most minerals, except for zinc, increased over time, with sodium, potassium, and magnesium reaching their highest levels after 15 min of cooking. FFAs also increased after 5 min of stir-frying, correlating with enhanced lipid oxidation. The electronic tongue and sensory evaluations confirmed the progressive increase in umami and saltiness, while bitterness and sourness remained minimal. PCA demonstrated that the first two principal components explained 79.18% of the variance, effectively differentiating samples by cooking time.

**Conclusion:**

This study provides novel insights into the mechanisms by which cooking duration affects flavor development in squid tentacles, emphasizing the critical roles of FAAs and FFAs. The findings suggest practical applications for optimizing cooking methods in the seafood industry to improve flavor quality and resource utilization.

## 1 Introduction

Stir-frying is a traditional Chinese cooking method characterized by the use of oil as the heat transfer medium to rapidly cook ingredients at high temperatures (1, 2). Compared to steaming or braising, stir-frying generates intense heat in a short period, effectively developing fresh, fragrant aromas and a crispy texture, making it widely popular (1, 3).

Taste perception is a multifaceted experience influenced by various compounds, including free amino acids (FAAs), fatty acids (FFAs), and minerals (4). The stir-frying method, a traditional Chinese cooking technique widely adopted in culinary practices around the world, is particularly effective at enhancing the flavors of ingredients due to its high-temperature, quick-cooking process (3). This method facilitates the Maillard reaction and other thermal reactions that contribute to the development of complex flavor profiles. While significant research has been conducted on flavor compounds in seafood, the focus has largely been on volatile compounds, with limited attention given to the specific contributions of FAAs and FFAs in non-volatile flavor development.

The Argentine squid (*Illex argentinus*) is an important seafood species, often processed for its body, while the tentacles are frequently discarded or underutilized (5). Tentacles contain ~2.14% more protein than the body and are rich in essential amino acids, making them a valuable yet overlooked resource in the seafood industry. Recent studies have highlighted the nutritional and culinary potential of squid tentacles, emphasizing their unique flavor attributes and the need for innovative processing techniques to enhance their marketability (6–8).

Despite the increasing interest in flavor chemistry and sensory evaluation within the seafood sector, there remains a significant gap in understanding how cooking methods, particularly stir-frying, influence the flavor development of squid tentacles. For example, studies have shown that the duration of cooking can significantly affect the composition of FAAs and FFAs, which are essential in determining taste attributes such as umami and richness (9–12). However, comprehensive studies examining the specific mechanisms by which cooking duration impacts these compounds in squid tentacles are lacking.

This study aims to address this gap by investigating the regulation mechanism of cooking duration on flavor compounds in squid tentacles. Focusing exclusively on the tentacles allows us to elucidate the dynamic contributions of FAAs, FFAs, and minerals to flavor development during stir-frying. The findings may provide valuable insights for optimizing cooking methods in the seafood industry, thereby enhancing the sensory quality and resource utilization of squid products.

## 2 Materials and methods

### 2.1 Materials

Frozen Argentine squid tentacles (Illex argentinus) were supplied by Zhoushan Marine Fisheries Co., Ltd. (Zhoushan, China) and stored at −18°C until use. Golden Dragon Fish edible vegetable blended oil was purchased from Zhejiang Yihai Kerry Food Industry Co., Ltd. (Hangzhou, China).

### 2.2 Preparation of argentine squid tentacles

Frozen Argentine squid tentacles were thawed at 4°C overnight before cooking. Thawed Argentine squid tentacles (200 g) were then placed in a wok preheated to 120°C over an open flame. A temperature of 120 °C and the use of a common blended vegetable oil were selected to represent typical household and small-scale industrial stir-frying practices for squid tentacle snacks in eastern China. Stir-frying commenced at this point (time zero). Samples were collected at 0, 2, 5, 10, and 15 min, designated as S1, S2, S3, S4, and S5, respectively. Continuous stirring ensured uniform heating. At each time point, subsamples were removed, rapidly cooled to room temperature, vacuum packed, and stored at −20 °C until chemical and sensory analyses. Unless otherwise specified, all chemical determinations at each stir-frying time were performed on three independently prepared samples, and data are expressed as mean ± standard deviation (*n* = 3).

It should be noted that only one temperature (120 °C) and one type of cooking oil (Golden Dragon Fish edible vegetable blended oil) were used in this study. This was carried out to standardize the cooking conditions and concentrate on the effects of cooking time on the flavor and nutrient content of squid tentacles. However, the use of different temperatures and oils could provide insights into the variability of these effects across a broader range of cooking conditions. Future studies will investigate the effects of varying cooking temperatures and oils on flavor development, mineral retention, and overall sensory attributes to provide a more comprehensive understanding of these factors.

Each cooking condition was replicated three times to ensure consistency and reliability of the results. The use of a single cooking condition (120 °C over an open flame) was based on standard preparation methods for squid, but future studies may consider varying cooking parameters (e.g., temperature, oil type, cooking method) to further explore their effects on the sensory and chemical properties of the squid tentacles.

### 2.3 Determination of mineral element content

The contents of sodium (Na), magnesium (Mg), potassium (K), iron (Fe), zinc (Zn), and calcium (Ca) were determined according to the Chinese National Standard GB 5009.268-2016 (Determination of multi-elements in food) (13). For each time point (S1–S5), mineral contents were measured in triplicate. The sample size for mineral analysis was set at *n* = 3 replicates for each time point (S1–S5). Each replicate was measured in triplicate to ensure the accuracy and reproducibility of the results.

### 2.4 Determination of free amino acid content

1 g of thoroughly crushed S1–S5 sample was mixed with 10 ml of 20% (w/v) trichloroacetic acid solution. After standing for 15 min, the mixture was centrifuged at 6,000 × g for 15 min. The supernatant was membrane-filtered (0.22 μm) and analyzed for FAA content using a chromatographic amino acid analysis system (L-8900, Hitachi, Tokyo, Japan) equipped with a cation-exchange column and post-column ninhydrin derivatization. Individual FAAs were identified and quantified by external calibration with mixed amino acid standards. FAA analyses were conducted in triplicate for each cooking time. For FAA analysis, a total of 3 replicates were performed at each sampling time point (S1–S5), with each replicate measured in triplicate to ensure precision and reliability of the data.

### 2.5 Electronic tongue analysis

Fifteen grams of each S1–S5 sample were weighed into a centrifuge tube, mixed with 30 ml of deionized water, and extracted at 45 °C for 15 min. After filtration, the volume was adjusted to 100 ml. The supernatant served as the test solution for electronic tongue analysis using an ASTREE II system (Alpha MOS, Toulouse, France). Before measurement, the sensor array was conditioned and calibrated according to the manufacturer's protocol using a standard reference solution. Each sample extract was measured in triplicate, and a reference solution was analyzed periodically to monitor sensor stability. The relative standard deviation of repeated measurements was kept below 10%, indicating acceptable reproducibility.

The electronic tongue system (ASTREE II, Alpha MOS, France) was used to analyze the taste characteristics of the squid tentacle samples. Prior to testing, the system underwent calibration using standard reference solutions for each taste attribute (umami, salty, bitter, sour) to ensure accurate measurements. Calibration was performed according to the manufacturer's instructions, with a series of known concentrations of taste standards (monosodium glutamate for umami, sodium chloride for salty, citric acid for sour, and lotus plumule for bitter). Calibration curves were generated, and the system was checked for consistency at regular intervals throughout the analysis. For validation, a subset of samples was analyzed in duplicate to assess the reproducibility of the electronic tongue readings. The reproducibility of the system was confirmed with a coefficient of variation (CV) of less than 5% for all test samples. The system was also subjected to a routine performance check using standard solutions before each experimental run to verify its operational consistency. The electronic tongue measurements were conducted in triplicate for each sample to ensure reliability. The results were averaged, and any discrepancies between replicate measurements were considered in the final data analysis.

### 2.6 Analysis of sensory taste characteristics

Sensory analysis was conducted following the method reported by Wang et al. (1). Thirteen trained panelists (seven females, six males, aged 22–35 years), familiar with squid tentacle taste characteristics, participated. Panelists were recruited and screened for the absence of seafood allergy and smoking. Four training sessions, each lasting ~60 min, were held to establish the basic taste attributes, reference standards, and intensity scales for squid tentacles, according to GB/T 29604-2013 (Sensory analysis—General guidelines for establishing sensory characteristic reference samples) (14). The final reference standards and corresponding sensory intensities are presented in Table 1. Panelists rated each attribute on a nine-point unstructured scale (1 = very weak, 9 = very strong). Ethical approval was not required for this study. The sensory evaluation was conducted in duplicate for each sample (S1–S5), and the data were averaged for analysis. Although the sample size of the sensory panel was relatively small, it was in line with typical practices in sensory studies involving specialized food products. Future studies should consider expanding the panel size and incorporating a broader demographic to improve the representativeness of the sensory data.

**Table 1:** Taste attributes and reference materials for sensory evaluation of squid tentacles.

**Taste attribute**	**Reference material**	**Concentration (g/100 ml)**	**Sensory intensity**
Umami	Monosodium glutamate	0.25	2
		1.00	5
Salty	Sodium chloride	0.50	4
		1.00	8
Bitter	Lotus plumule	0.05	2
		0.60	6
Sour	Citric acid	0.08	5
	Glutathione	0.09	5

### 2.7 Determination of free fatty acid content

Fatty acid content was determined according to the Chinese National Standard GB 5009.168-2016 (Determination of fatty acids in food) (14). Lipids were extracted and converted to fatty acid methyl esters (FAMEs) following this standard. FAMEs were analyzed on a gas chromatograph (CP3800, Agilent Technologies Inc., California, America) equipped with a flame ionization detector and a polar capillary column (for example, 30 cm × 0.25 mm internal diameter, 0.25 μm film thickness). The injector and detector temperatures and oven temperature program followed GB 5009.168-2016 (14). Individual free fatty acids were identified by comparing retention times with those of a mixed FAME standard (Sigma, cas9150-89-4) and quantified using external calibration. FFA analyses were also performed in triplicate for each treatment.

Free fatty acid content was analyzed with three replicates for each time point (S1–S5). Each replicate was measured in triplicate to minimize measurement error and enhance the reliability of the results.

### 2.8 Statistical analysis

Statistical analyses were performed using SPSS 23.0 software (IBM Corp., Armonk, NY, USA). For comparing differences between treatment groups, a more robust statistical method, such as Tukey's HSD (Honestly Significant Difference) test, was used to reduce the risk of false positives compared to the previously applied Least Significant Difference (LSD) method. Tukey's test was selected due to its greater control over Type I error rates when performing multiple comparisons.

In addition to the primary analysis, *post-hoc* corrections (e.g., Bonferroni adjustment) were applied where appropriate to account for multiple comparisons and further reduce the likelihood of type I errors.

Principal Component Analysis (PCA) was used to assess the relationships between various sensory attributes and chemical components of the squid tentacles. To ensure the validity of the PCA, the Kaiser-Meyer-Olkin (KMO) measure of sampling adequacy and Bartlett's test of sphericity were performed prior to analysis. KMO values greater than 0.6 and a significant result from Bartlett's test (*P* < 0.05) confirmed the appropriateness of using PCA for this dataset. Data were considered statistically significant at a significance level of *P* < 0.05. All statistical analyses were conducted in triplicate to ensure reliability and reproducibility of the results.

For comparing differences between treatment groups, Tukey's honestly significant difference (HSD) test was used, which is more robust than the least significant difference (LSD) test and reduces the risk of false positives in multiple comparisons. Additionally, Bonferroni corrections were applied to further adjust for the multiple comparisons and control for type I errors.

For principal component analysis (PCA), the Kaiser-Meyer-Olkin (KMO) measure of sampling adequacy and Bartlett's test of sphericity were performed to ensure the suitability of the data for PCA. A KMO value greater than 0.6 and a significant result from Bartlett's test (*P* < 0.05) confirmed the validity of the PCA approach. All statistical analyses were conducted in triplicate to ensure the reproducibility and reliability of the findings.

## 3 Results and discussion

### 3.1 Dynamic changes in mineral element content

**Figure 1:**
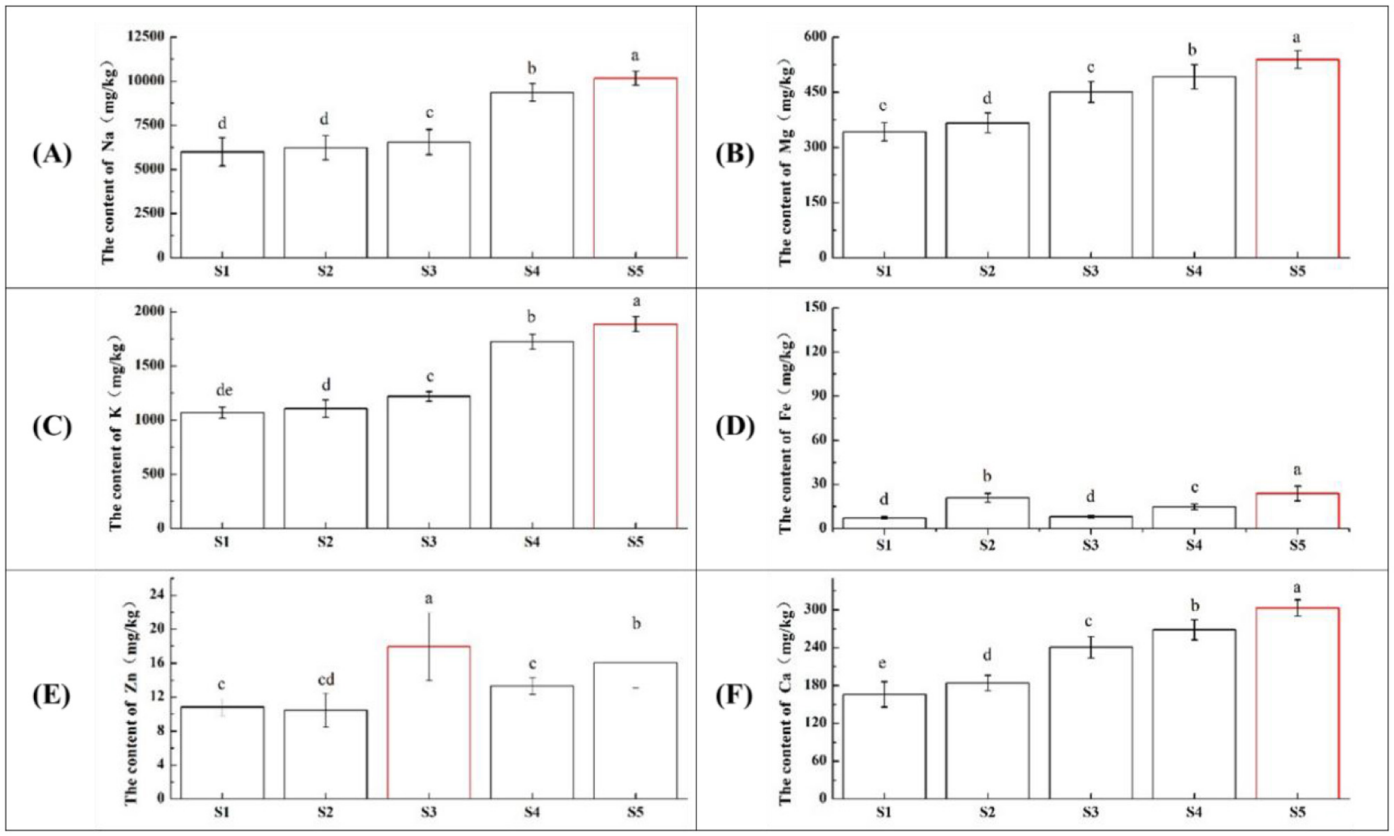
Dynamic changes in mineral element content of squid tentacles during traditional stir-frying. **(A)** Na; **(B)** Mg; **(C)** K; **(D)** Ca; **(E)** Fe; **(F)** Zn. S1–S5 correspond to stir-frying times of 0, 2, 5, 10, and 15 min, respectively. Different lowercase letters denote significant differences among treatments (*P* < 0.05).

Mineral elements maintain bone structure, regulate acid-base balance, and function in hormones, enzymes, and enzyme activation (15). The content of six mineral elements in squid tentacles during stir-frying is shown in Figure 1. Na, Mg, and K were the most abundant minerals, followed by Ca, with Fe and Zn present in lower amounts. Levels of Na, K, Mg, Ca, and Fe generally increased with stir-frying time, peaking at S5. Na was predominant, reaching 1.0 × 104 mg/kg at S5, contributing to vitality and metabolism. K content exceeded 1.0 × 103 mg/kg across all samples, higher than eggs (600 mg/kg) but lower than beef (3.3 × 103 mg/kg) (16), playing a role in nervous system regulation. Mg is essential for nerve and muscle function and blood sugar conversion (17). Fe and Zn, the most abundant trace elements in squid tentacles, are vital for human health (18). Unlike other minerals, Zn content peaked at S3 (Figure 1), potentially due to loss during prolonged high-temperature stir-frying as moisture migrated to the cooking medium. Therefore, shorter high-temperature cooking may help minimize mineral loss (19).

In terms of mineral content, the increase in sodium and potassium levels with stir-frying is comparable to findings in other seafood species, such as shrimp, where similar trends were found during high-temperature cooking processes (20). This further supports the notion that seafood can be a rich source of essential minerals, particularly sodium and potassium, which are vital for human health and flavor perception (21).

### 3.2 Dynamic changes in free amino acid content

Free amino acids (FAAs), the end products of protein hydrolysis, impart unique texture and serve as critical flavor precursors. Their levels during processing reflect the balance between formation and degradation rates. As shown in Table 2, protein hydrolysis increased notably after the S3 stage (5 min), with the formation rate of most FAAs exceeding degradation, leading to continuous accumulation. Levels of key umami amino acids, glutamic acid and aspartic acid, remained high and increased progressively, likely underpinning the characteristic delicious taste of stir-fried squid tentacles.

**Table 2:** Changes in free amino acid content (g/100 g) of squid tentacles during traditional stir-frying (S1: 0 min, S2: 2 min, S3: 5 min, S4: 10 min, S5: 15 min).

**Amino acid**	**Type**	**S1**	**S2**	**S3**	**S4**	**S5**
Aspartic acid (Asp)⋆	Umami	1.15 ± 0.02^c^	1.43 ± 0.00^b^	1.16 ± 0.01^c^	1.94 ± 0.03^a^	1.92 ± 0.04^a^
Threonine (Thr)^*^	Essential	0.52 ± 0.01^c^	0.92 ± 0.06^b^	0.32 ± 0.00^d^	1.27 ± 0.03^a^	0.87 ± 0.03^bc^
Serine (Ser)		0.57 ± 0.02^b^	0.39 ± 0.01^c^	0.52 ± 0.03^b^	0.56 ± 0.00^b^	0.89 ± 0.03^a^
Glutamic acid (Glu)⋆	Umami	2.08 ± 0.03^c^	2.60 ± 0.02^b^	2.21 ± 0.03^bc^	3.43 ± 0.06^a^	3.55 ± 0.00^a^
Glycine (Gly)⋆	Umami	1.13 ± 0.01^a^	0.66 ± 0.03^b^	0.64 ± 0.04^b^	1.02 ± 0.03^a^	1.11 ± 0.06^a^
Alanine (Ala)⋆	Umami	0.68 ± 0.00^c^	0.75 ± 0.03^b^	0.64 ± 0.02^c^	1.06 ± 0.03^a^	1.04 ± 0.03^a^
Valine (Val)^*^	Essential	0.49 ± 0.04^c^	0.61 ± 0.06^b^	0.50 ± 0.03^c^	0.85 ± 0.00^a^	0.83 ± 0.02^a^
Methionine (Met)^*^	Essential	0.36 ± 0.03^c^	0.44 ± 0.04^b^	0.35 ± 0.03^c^	0.64 ± 0.03^a^	0.59 ± 0.04^ab^
Isoleucine (Ile)^*^	Essential	0.52 ± 0.03^d^	0.71 ± 0.06^c^	0.56 ± 0.02^d^	1.01 ± 0.05^a^	0.92 ± 0.01^b^
Leucine (Leu)^*^	Essential	0.87 ± 0.00^c^	1.16 ± 0.02^b^	0.91 ± 0.06^c^	1.61 ± 0.03^a^	1.56 ± 0.02^ab^
Tyrosine (Tyr)		0.40 ± 0.03^c^	0.38 ± 0.01^c^	0.33 ± 0.03^d^	0.64 ± 0.04^a^	0.55 ± 0.03^b^
Phenylalanine (Phe)^*^	Essential	0.49 ± 0.01^c^	0.63 ± 0.03^b^	0.49 ± 0.00^c^	0.89 ± 0.06^a^	0.80 ± 0.01^a^
Lysine (Lys)^*^	Essential	0.79 ± 0.03^d^	1.20 ± 0.04^b^	0.90 ± 0.03^c^	1.49 ± 0.00^ab^	1.57 ± 0.05^a^
Histidine (His)		0.27 ± 0.02^d^	0.35 ± 0.03^c^	0.24 ± 0.01^d^	0.41 ± 0.03^b^	0.51 ± 0.04^a^
Arginine (Arg)		0.86 ± 0.01^c^	1.06 ± 0.06^b^	0.79 ± 0.02^d^	1.43 ± 0.06^ab^	1.48 ± 0.01^a^
Proline (Pro)		0.37 ± 0.02^b^	0.28 ± 0.03^c^	0.16 ± 0.01^d^	0.35 ± 0.03^b^	0.42 ± 0.00^a^
Total FAAs		11.5 ± 0.03^c^	13.6 ± 0.01^b^	10.7 ± 0.02^d^	18.6 ± 0.02^a^	18.6 ± 0.04^a^
Essential FAAs^*^		4.03 ± 0.01^d^	5.66 ± 0.02^c^	4.04 ± 0.00^d^	7.74 ± 0.03^a^	7.14 ± 0.02^b^
Umami FAAs⋆		7.51 ± 0.01^d^	7.89 ± 0.01^c^	6.69 ± 0.03^e^	10.8 ± 0.02^b^	11.5 ± 0.01^a^

^*^Data are mean ± SD (*n* = 3). Different lowercase letters within the same row indicate significant differences (*P* < 0.05). ^*^ denotes essential amino acids.

**Figure 2:**
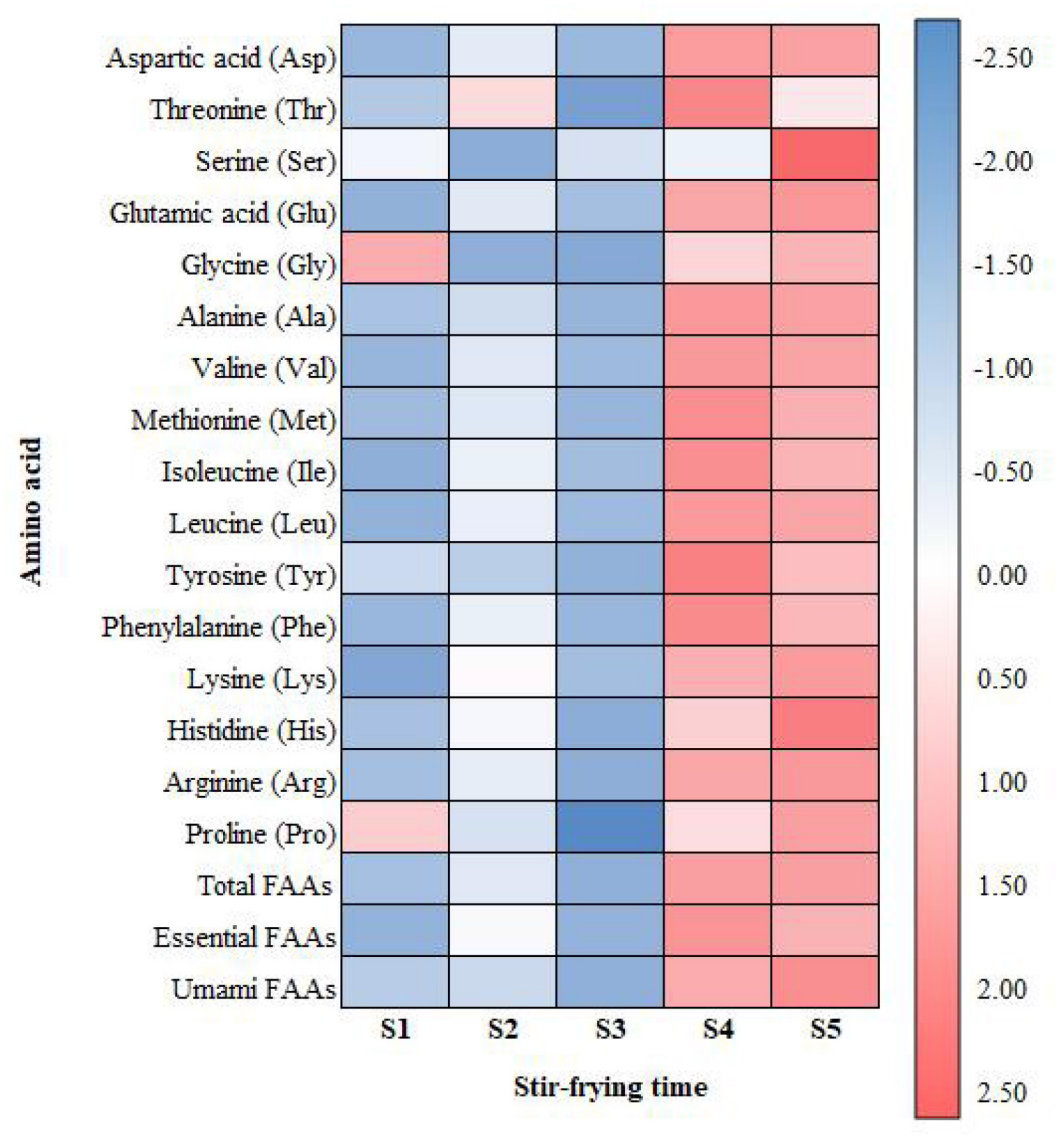
Heatmap of relative changes in free amino acids (FAAs) of squid tentacles during traditional stir-frying. Values represent *z*-score standardized concentrations across samples (S1–S5: 0, 2, 5, 10, and 15 min).

FAA accumulation provides a rich substrate for the Maillard reaction. Methionine, a precursor for sulfurous volatile compounds, contributes salty, meaty, and soy sauce-like flavors (22). Alanine, glycine, serine, and threonine can produce pleasant caramel notes via Maillard reaction and undergo Strecker degradation to form aldehydes, ultimately yielding aromatic pyrazines (23, 24). Valine, leucine, isoleucine, and phenylalanine are key precursors for branched-chain and aromatic aldehydes (25, 26). These findings suggest that flavor development in squid tentacles involves complex cumulative, synergistic, and interactive effects of thermal reaction products rather than simple additive accumulation. To aid interpretation, the relative changes in individual FAAs across stir-frying times are also visualized as a heatmap (Figure 2).

Similar time-dependent increases in umami FAAs and related Maillard precursors have been reported in heated squid mantle, fish sauce, and other seafood systems, where processing promotes proteolysis and *de novo* formation of taste-active peptides and amino acids. These results support the view that squid tentacles, which are rich in protein and FAAs, are particularly susceptible to Maillard browning and the generation of complex savory notes during stir-frying.

The observed changes in FAA content during stir-frying are consistent with findings from other seafood studies, where thermal processing also promoted the accumulation of umami precursors, such as glutamic acid and aspartic acid (27, 28). Similar research on fish and shrimp has demonstrated that the breakdown of proteins into FAAs contributes significantly to the enhancement of savory flavors during heat treatment (29–31).

This study's results align with reports on meat flavor development, where Maillard reaction products, particularly amino acid-derived volatiles, play a central role in the generation of meaty and umami flavors during cooking (32, 33). The accumulation of essential amino acids, such as valine and leucine, in squid tentacles during stir-frying mirrors patterns found in beef and pork, suggesting that these compounds are universally important for flavor formation in both aquatic and terrestrial meats.

### 3.3 Electronic tongue and sensory evaluation of taste quality

#### 3.3.1 Electronic tongue analysis of taste attributes

**Figure 3:**
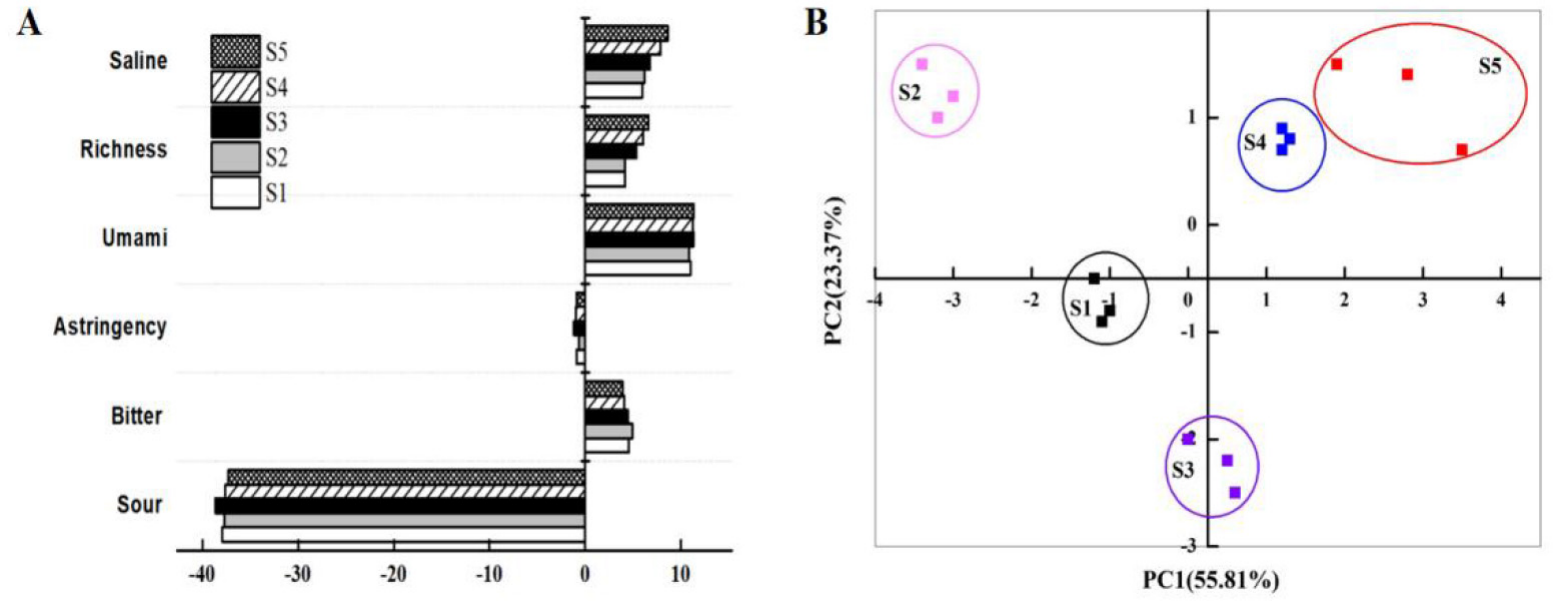
The taste attributes of the electronic tongue **(A)** and PCA analysis **(B)** of squid tentacles during traditional stir-frying.

The electronic tongue characterized taste changes during stir-frying (Figure 3A). Umami richness (aftertaste of umami compounds) increased continuously, indicating gradual degradation and accumulation of taste compounds. The bitter-sour response, associated with long-chain peptides, initially increased but then decreased in later stages (S4–S5), suggesting degradation of large peptides. Saltiness increased progressively, attributable to water loss during cooking and the inherent salty contribution of accumulated umami compounds. Similar time-dependent increases in umami and saltiness have been reported in stir-fried and dry-cured meat systems, where proteolysis and moisture loss concentrate taste-active compounds and enhance Maillard-derived flavors (34).

Principal component analysis (PCA) of the electronic tongue data (Figure 3B) revealed that the first two principal components (PC1 and PC2) explained 79.18% of the total variance (PC1: 55.81%, PC2: 23.37%), indicating effective capture of sample taste characteristics. The electronic tongue clearly discriminated between samples at different heating stages. Samples S4 and S5 clustered closely, suggesting taste quality stabilized during the later stages of heating.

#### 3.3.2 Sensory analysis of taste attributes

**Figure 4:**
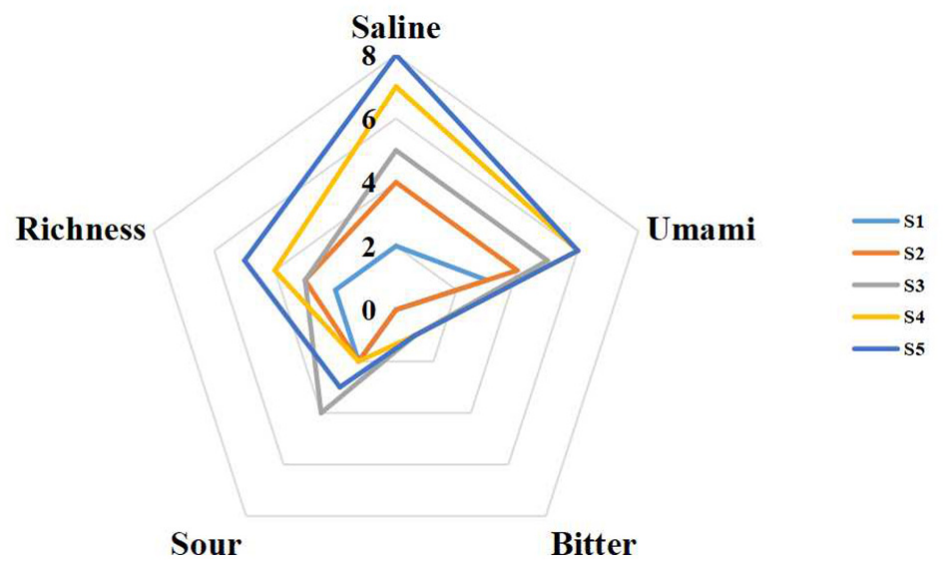
Difference in sensory taste characteristics of squid tentacles during traditional stir-frying.

While electronic tongues mimic human taste perception, taste is determined by complex chemical, physicochemical, and biological factors, and their results cannot fully replace human sensory evaluation, especially for solid foods where matrix effects are significant. Sensory analysis results (Figure 4) showed saltiness progressively increased from S1 to S5, aligning with the electronic tongue. Perceived sourness and bitterness scores were low, indicating subtle levels difficult for panelists to detect post-stir-frying. The higher sour response detected by the electronic tongue might reflect its sensitivity to specific compounds, whereas sensory perception is modulated by matrix effects and compound dissolution. These patterns are consistent with previous observations that increasing sodium chloride levels and accumulation of umami amino acids during thermal processing enhance perceived saltiness and umami while maintaining low sensory intensity for sourness and bitterness (35–37). Studies on ham, surimi, and other seafood products have likewise shown that matrix properties and fat content modulate how chemical composition translates into perceived taste (38, 39). Recent studies using combined electronic tongue and sensory approaches in fish cakes, fish soup, and tea have likewise demonstrated that processing conditions and matrix composition can differentially affect instrumental taste signals and human perception, underscoring the need for integrated interpretation.

While the electronic tongue can mimic human taste perception, the sensory perception of taste in solid foods is influenced by complex matrix effects, which involve the interactions between food components (such as fat, protein, and water) and the taste compounds. These matrix effects can alter the release, solubility, and volatility of specific compounds, leading to discrepancies between electronic tongue data and human sensory evaluation. For example, the electronic tongue may detect compounds in a homogenized liquid extract, whereas in actual food consumption, the sensory perception is shaped by how these compounds are released and perceived by the taste receptors in the oral cavity. This can explain the higher sourness detected by the electronic tongue compared to the low sourness scores reported by panelists. Furthermore, the electronic tongue sensors are more sensitive to certain compounds, especially small molecules, such as amino acids and salts, which can be more readily detected in liquid extracts, while human sensory perception may be more influenced by the overall texture and mouthfeel of the food, which the electronic tongue cannot fully simulate.

#### 3.3.3 Correlation between sensory properties and electronic tongue

**Figure 5:**
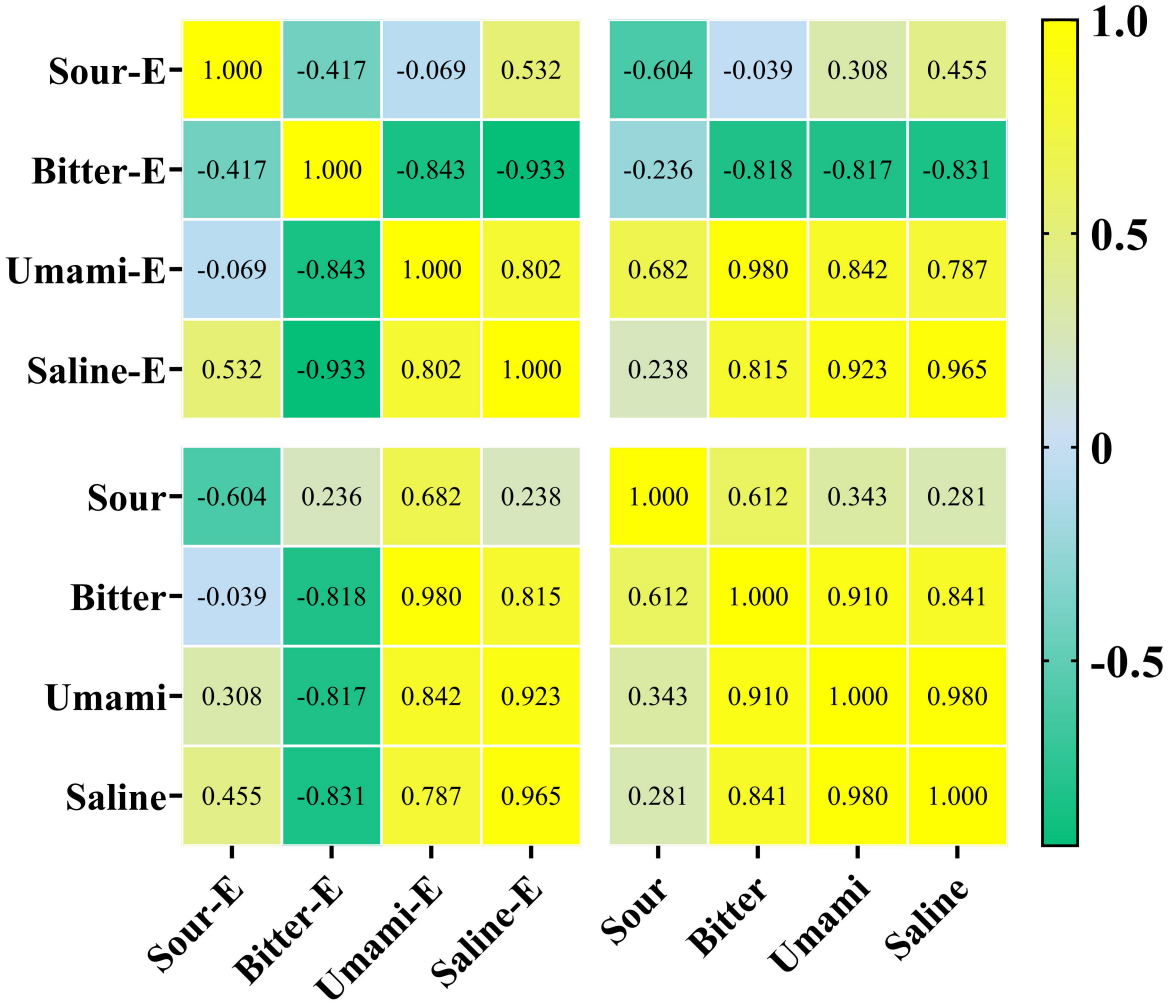
The heatmap of the taste attributes of electronic tongue and sensory analysis. The suffix E represents the taste attribute results from electronic tongue analysis.

Correlation analysis between sensory scores and electronic tongue responses (Figure 5) revealed complexity. Sensory bitterness was negatively correlated with electronic tongue bitterness. Inconsistencies existed, for example, electronic tongue umami (E_Umami) correlated positively with sensory umami but negatively with sensory sourness. This discrepancy likely arises because electronic tongue sensors target specific analytes in homogenized, often fat-depleted liquid extracts, whereas actual food consumption involves significant matrix effects crucial for sensory quality. Therefore, while useful for rapid screening, electronic tongue results for solid foods should be interpreted alongside sensory evaluations. Comparable discrepancies between instrumental and sensory responses have been documented for meats, hams, surimi, and fish products (40, 41), which reinforces the need to interpret electronic tongue measurements in the context of product-specific matrix effects.

The observed discrepancies between the electronic tongue and sensory data can be attributed to the different methods of analysis: the electronic tongue evaluates analytes from homogenized samples, which may not accurately reflect how these compounds behave in a more complex food matrix during actual consumption. Sensory evaluations, on the other hand, are influenced by factors, such as food texture, temperature, and the matrix's ability to release specific compounds, which electronic tongues cannot replicate (42, 43). For example, the negative correlation between electronic tongue umami and sensory sourness is likely due to the fact that umami compounds are often associated with glutamic acid and nucleotides, which are more soluble and detectable in liquid extracts. In contrast, sourness, which is primarily influenced by organic acids and is more dependent on the matrix, may not be perceived as strongly in solid foods (44).

### 3.4 Dynamic changes in free fatty acid content

**Figure 6:**
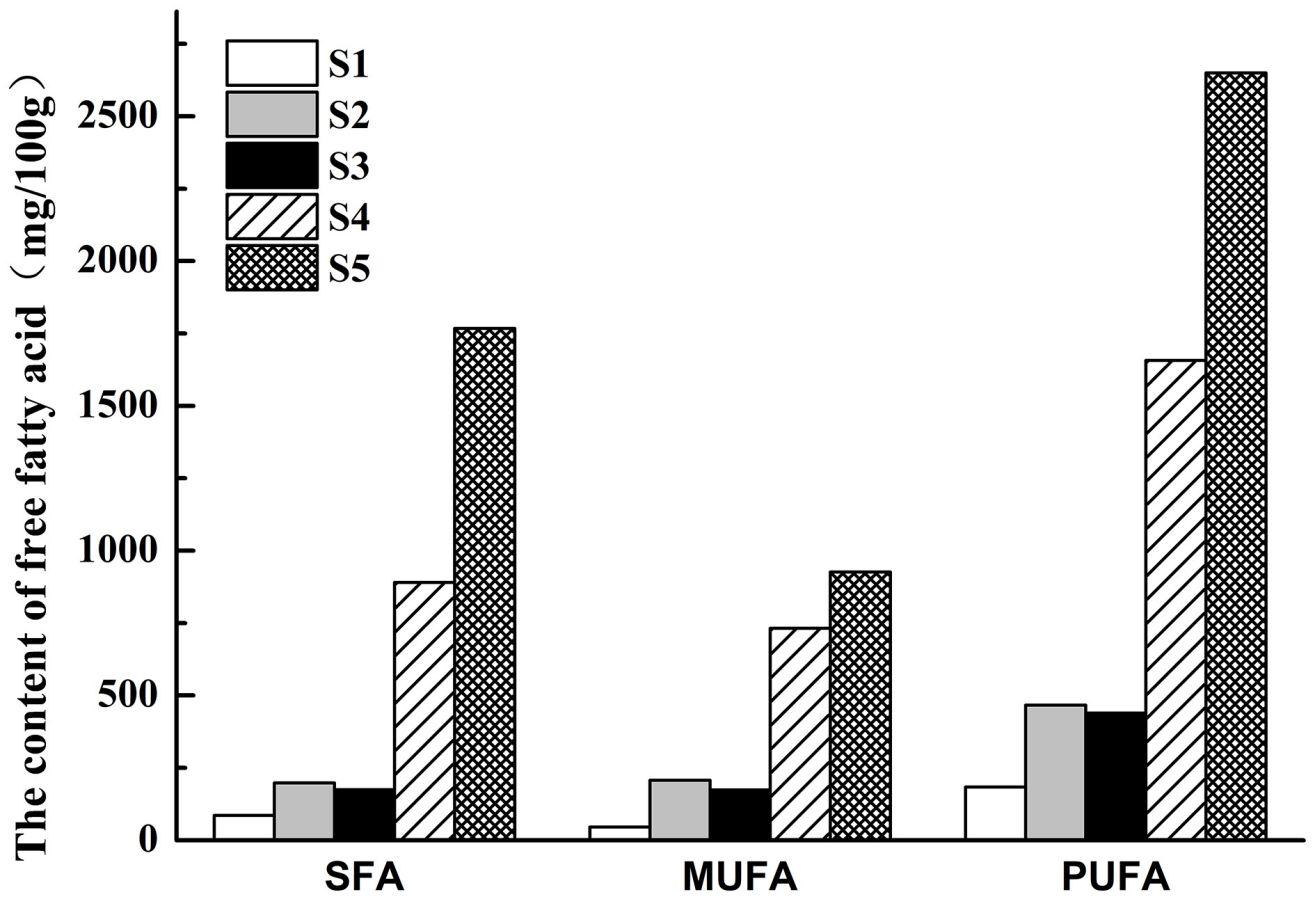
The content changes of free fatty acids of squid tentacles during traditional stir-frying.

Free fatty acids (FFAs), while tasteless, influence texture richness and oxidize to produce key volatile flavor compounds (aldehydes, alcohols, ketones). Stir-frying duration significantly impacted FFA content and composition in squid tentacles, primarily due to varying degrees of lipid oxidation, affecting both quality and nutritional value. Stir-frying thus comprehensively reflects squid tentacle flavor development. The process accelerates oxidation and loss of unsaturated fatty acids, potentially diminishing nutritional quality. FFA levels initially decreased from S1 to S2 (Figure 6), likely due to accelerated oxidation converting FFAs into secondary products and volatiles. Subsequently, from S3 onwards, FFA accumulation significantly increased, indicating enhanced fat hydrolysis as stir-frying progressed, consistent with previous findings (45).

The initial decline, followed by the accumulation of FFAs observed here is consistent with the interplay between lipid oxidation and hydrolysis during thermal processing of seafood and meat. Polyunsaturated fatty acids can be rapidly oxidized at the onset of heating, forming volatile aldehydes and other secondary products, whereas prolonged cooking promotes continued hydrolysis of glycerides and phospholipids, increasing the pool of FFAs. Similar patterns have been reported for squid mantle and crab, highlighting the trade-off between maintaining nutritional quality (n-3 PUFAs) and generating desirable aroma compounds at higher temperatures and longer times.

The changes in FFA content observed in squid tentacles during stir-frying align with studies on fatty fish, where stir-frying or grilling induced a noticeable increase in FFAs, which contributed to the formation of aldehydes and other volatile compounds that enhance flavor complexity. Such patterns have been found in studies of other marine foods, including mackerel, where the oxidation of polyunsaturated fatty acids is linked to flavor development, although with some nutritional trade-offs.

The synergistic interactions among FAAs, fatty acids, and other thermal reaction products found in squid tentacles mirror those documented in meat flavor studies, where these compounds not only accumulate but also interact in complex ways to produce rich, multifaceted flavors. Similar interactions have been explored in various fish species, highlighting the importance of considering these cumulative effects rather than simple additive accumulation in flavor development (46).

The findings of this study contribute to a growing body of research on seafood flavor development, which has been underexplored compared to terrestrial meats. Further comparative studies on different cooking methods, such as grilling or steaming, and their impact on flavor development in both squid tentacles and body meat could yield valuable insights into how processing conditions affect flavor profiles across various squid parts. Additionally, advanced techniques such as flavoromics and the use of consumer sensory panels could further enhance our understanding of how these flavors are perceived by different populations and inform seafood processing strategies.

### 3.5 Limitations of the study

While this study provides valuable insights into the flavor development of squid tentacles during stir-frying, several limitations should be acknowledged. The experiments were conducted under controlled laboratory conditions, which may not accurately reflect real-world cooking scenarios. Variations in cooking techniques, equipment, and environmental factors (such as humidity and altitude) can significantly influence flavor development and should be explored in future studies. This study concentrated primarily on free amino acids (FAAs), free fatty acids (FFAs), and minerals. Other important flavor compounds, such as volatile aromatic compounds and non-volatile substances, were not analyzed. A more comprehensive approach that includes these additional compounds could provide a fuller understanding of flavor dynamics. The sensory evaluation of the Argentine squid tentacles was conducted using 13 trained panelists. Although this number is consistent with previous sensory studies on seafood, it should be noted that the relatively small sample size may limit the generalizability and statistical reliability of the sensory results. Future studies should consider increasing the number of participants to enhance the robustness of sensory data and provide more reliable conclusions regarding the flavor profile and overall sensory characteristics of the squid tentacles.

## 4 Conclusions

This study provided novel insights into the formation of distinctive flavors in squid tentacles during traditional stir-frying, highlighting the complex interaction of flavor compounds, thermal reactions, and matrix effects. The accumulation of free amino acids, particularly umami precursors, was identified as a key driver in enhancing flavor richness, while polyunsaturated fatty acids contributed not only to the nutritional profile, but also to the formation of odor-active compounds. The findings indicated that flavor development in squid tentacles is driven by complex synergistic interactions among amino acids, fatty acids, and other thermal reaction products, rather than being a simple additive process. One of the unique contributions of this study lies in demonstrating how variations in stir-frying time impact the generation and accumulation of these flavor compounds, providing a deeper understanding of how cooking processes can influence the flavor profile of aquatic products. However, this study is not without limitations. The narrow cooking conditions (single stir-frying temperature and method), the small sensory panel, and the limited number of replicates must be acknowledged as constraints that warrant further investigation. These factors may limit the generalizability of the findings, and future studies should aim to incorporate more diverse cooking methods, longer or more varied sensory panels, and increased replicates for improved statistical robustness.

From a practical perspective, the results of this study can be applied to optimize cooking processes in seafood processing, with potential implications for improving flavor consistency in commercial squid products. Understanding the dynamics of flavor compound formation can help in developing targeted strategies for flavor enhancement or modification, and guide the utilization of by-products, such as squid tentacles, in value-added products.

Looking ahead, there are several directions for extending this research. Future studies should explore different cooking techniques (e.g., grilling, boiling, or steaming) and temperatures to assess their effects on flavor development in squid tentacles and other seafood products. Comparing squid tentacles with body meat could provide further insights into whether similar flavor mechanisms operate across different parts of the squid. In addition, more advanced techniques in volatile/flavoromics analysis may provide a deeper understanding of the key aromatic compounds responsible for the sensory experience. Expanding sensory evaluations to include larger consumer panels may help refine understanding of how these flavors are perceived across different demographic groups, outlining valuable insights for both industry and consumers.

## Data Availability

The original contributions presented in the study are included in the article/supplementary material, further inquiries can be directed to the corresponding author.
